# Supplementary figures and images for: Construction of a de novo assembly pipeline using multiple transcriptome data sets from Cypripedium macranthos (Orchidaceae)
Source: PLoS One. 2023 Jun 6;18(6):e0286804. doi: 10.1371/journal.pone.0286804 (PMC10243633; doi:10.1371/journal.pone.0286804)

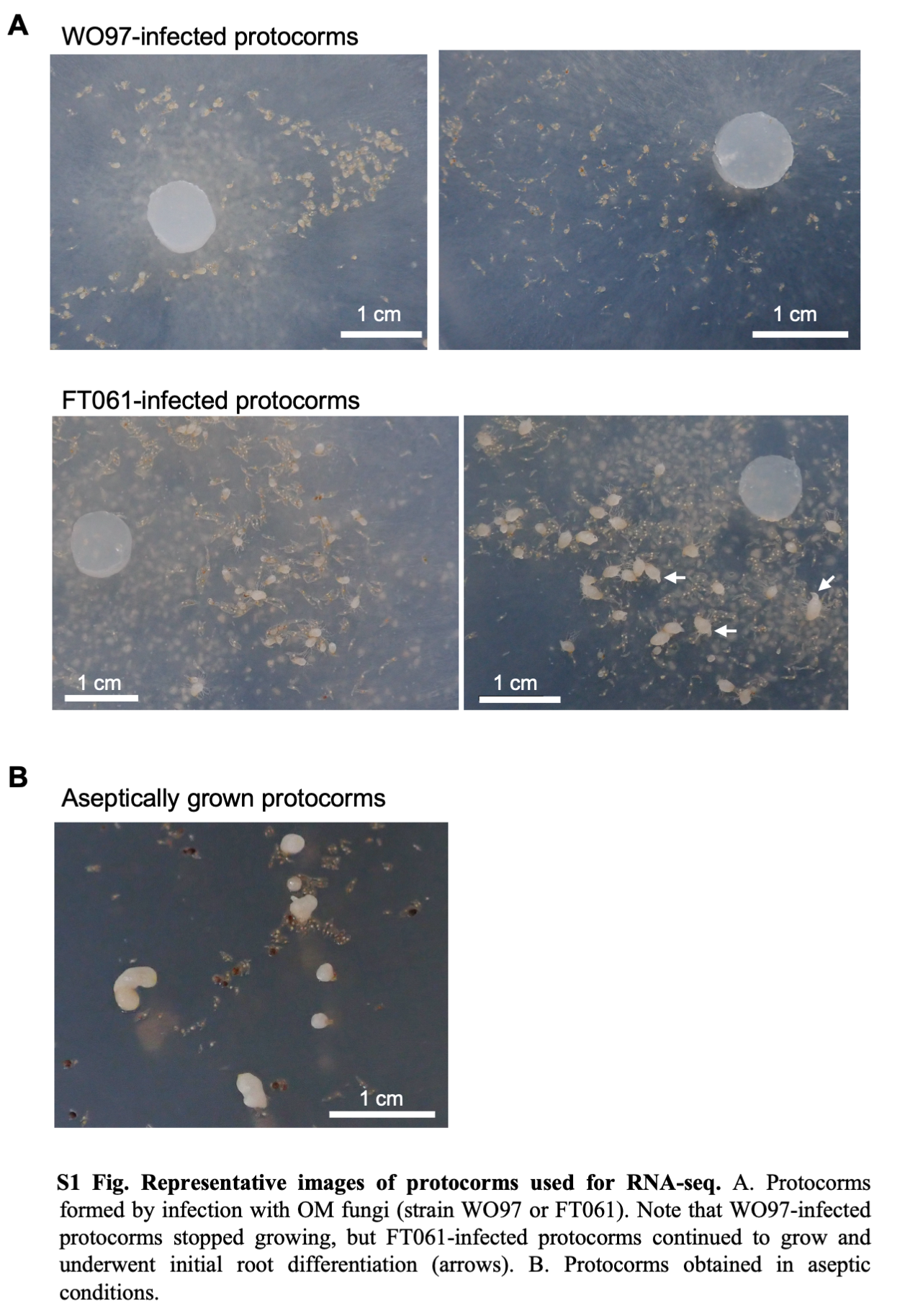

Supplement: S1 Fig — A. Protocorms formed by infection with OM fungi (strain W097 or FT061). Note that W097-infected protocorms stopped growing, but FT061-infected protocorms continued to grow and underwent initial root differentiation (arrows). B. Protocorms obtained in aseptic conditions. (TIF) [file pone.0286804.s001.tif]

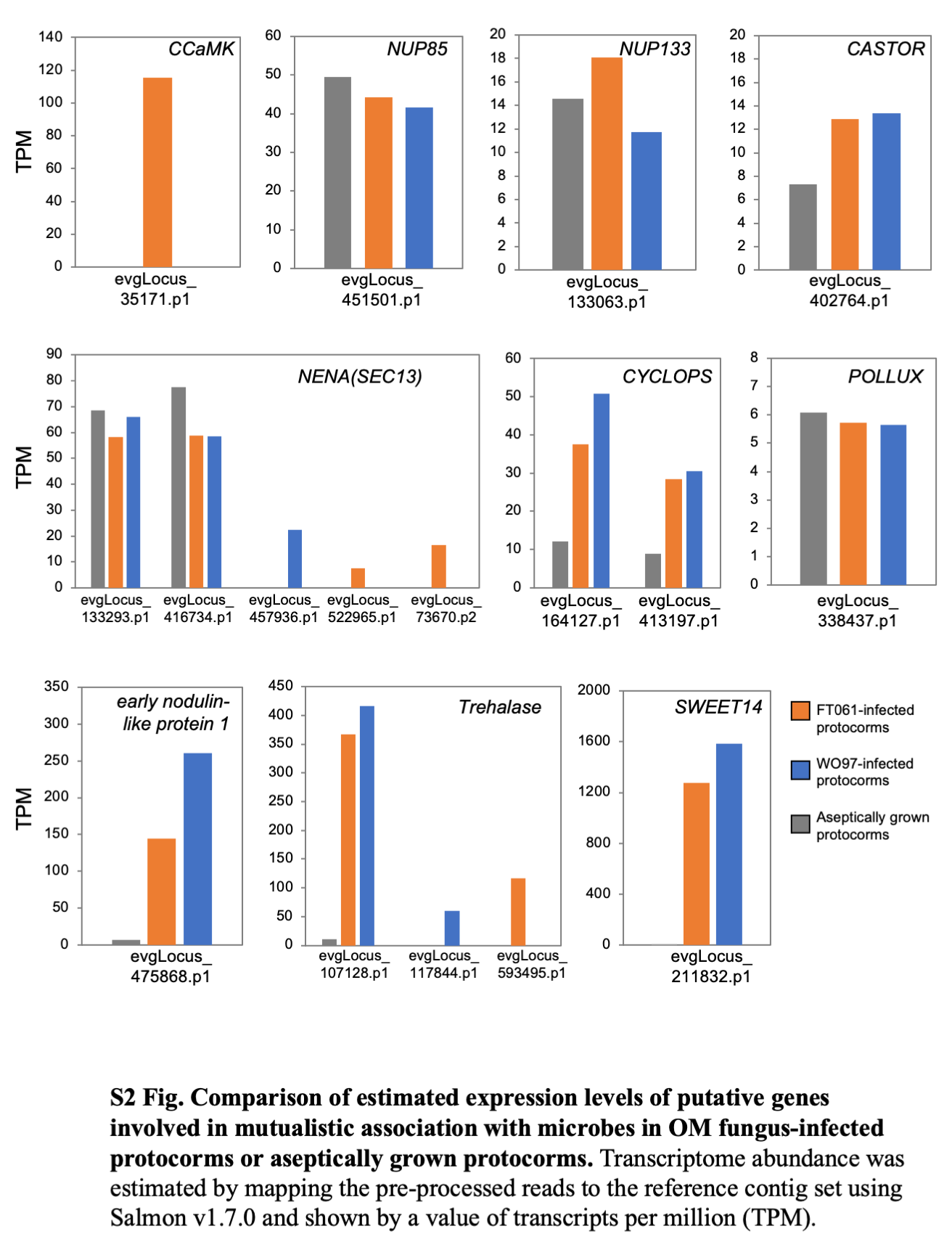

Supplement: S2 Fig — Transcriptome abundance was estimated by mapping the pre-processed reads to the reference contig set using Salmon vl .7.0 and shown by a value of transcripts per million (TPM). (TIF) [file pone.0286804.s002.tif]

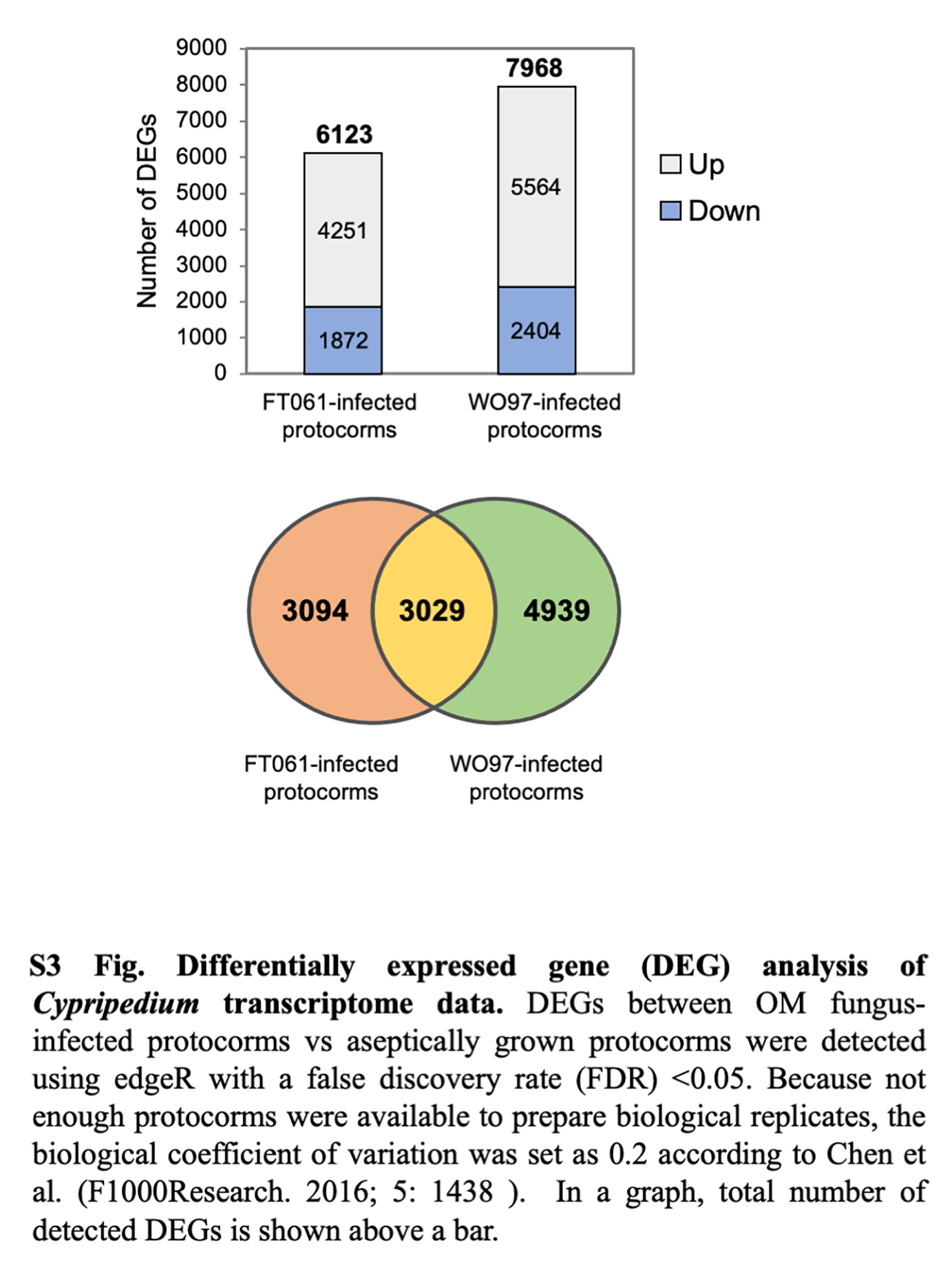

Supplement: S3 Fig — DEGs between OM fungus- infected protocorms vs aseptically grown protocorms were detected using edgeR with a false discovery rate (FDR) <0.05. Because not enough protocorms were available to prepare biological replicates, the biological coefficient of variation was set as 0.2 according to Chen et al. (FlOOOResearch. 2016; 5: 1438). In a graph, total number of detected DEGs is shown above a bar. (TIF) [file pone.0286804.s003.tif]

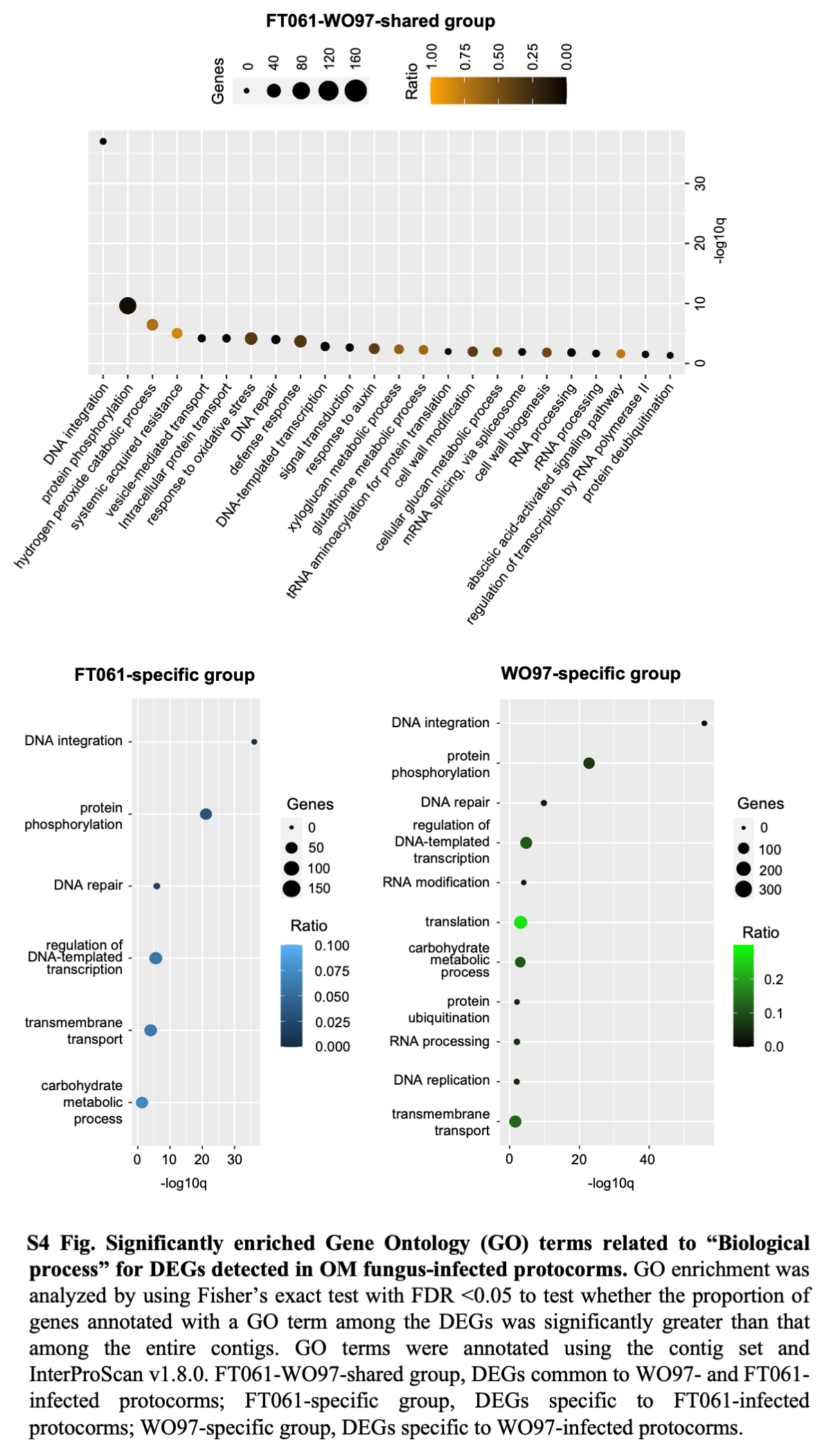

Supplement: S4 Fig — GO enrichment was analyzed by using Fisher’s exact test with FDR <0.05 to test whether the proportion of genes annotated with a GO term among the DEGs was significantly greater than that among the entire contigs. GO terms were annotated using the contig set and InterProScan vl.8.0. FT061-WO97-shared group, DEGs common to W097- and FT061- infected protocorms; FT061-specific group, DEGs specific to FT061-infected protocorms; W097-specific group, DEGs specific to W097-infected protocorms. (TIF) [file pone.0286804.s004.tif]
