# Supplementary material for: Construction of a de novo assembly pipeline using multiple transcriptome data sets from Cypripedium macranthos (Orchidaceae)
Source: PLoS One. 2023 Jun 6;18(6):e0286804. doi: 10.1371/journal.pone.0286804 (PMC10243633; doi:10.1371/journal.pone.0286804)
Supplement: Attachment — Submitted filename: Kambara_Response to Reviewers.docx [file pone.0286804.s008.docx]

Additional Editor Comments:

Besides the comments made by the reviewer, I also have the following observations regarding the manuscript:

1. Several typos occur in the text; the English could use a little bit of improvement.

>> Response by authors

Thank you for pointing this out. We carefully checked the text and found some typos; they are now corrected.

2. Please define basic terms, such as protocorm.

>> Response by authors

According to the suggestion, we added sentences to define the term ‘protocorm’. We also added sentences for ‘mapping rate’ according to a suggestion by Reviewer 1.

“In the process of germination, the embryo swells in size and develops into a protocorm before becoming a plantlet. Protocorm is a unique structure designed to establish an association with OM fungi.” (Lines 51-53)

“Mapping rate is defined as percentage of reads aligned (mapped) to the constructed contigs among all reads” (Lines 153-154)

3. Lines 21-23: “The family Orchidaceae comprises the most species of any monocotyledonous family and has interesting characteristics such as seed germination induced by mycorrhizal fungi and flower morphology that co-evolved with pollinators.” Is incorrect, it is more accurate to say that the fungi and the flower morphology co-adapted with pollinators. We are talking about already existing structures that underwent adaptation.

>> Response by authors

Thank you for advice. We changed the word “co-evolved” to “co-adapted”. (Line 23)

4. Line 190: table 1: incomplete value: 1048,92

>> Response by authors

We’re sorry for the mistake. We corrected the value 1048,92 to 104,892 in Table 1.

5. Why did the VL assembler have such a low mapping rate in data sets 1 and 3? Does this nit skew the results? Shouldn’t this assembler be excluded? Would the results change that much?

>> Response by authors

Thank you for asking the questions. As you pointed out, the assemblies constructed by Velvet (VL) had lower mapping rates and complete BUSCO % than the others. However, in some cases, the evaluation of assemblies by VL was comparable to others, i.e., assembly construction by VL seemed to exhibit unstable behavior. The VL is an assembler developed for genome assembly and has high performance for resolving repeat sequences; VL is not supposed for the use in *de novo* assembly of transcriptome data. On the other hand, the other four assemblers (Trinity, rnaSPAdes, Trans-ABySS and IDBA-Tran) were developed for *de novo* transcriptome assembly. At this point in time, we do not know the reason why some assemblies made in VL are inferior to others, but we think that one of the reasons is that the computational algorithm of VL is designed for genome construction with a high stability for repeated sequences. Nevertheless, VL is sometimes applied for analysis of transcriptome data as shown in Nakasugi et al. (2014 PLoS ONE, 9: e91776), and thus we also used VL to compare several assemblies derived from different assemblers. The assembly selected as the best assembler in this study is Assembly 4 (Trinity and IDBA-Tran) and exclusion of VL from our analysis did not significantly change our results. It can be said that VL is unsuitable for use in *de novo* transcriptome assembly in some cases, and we included additional sentences in the Discussion section. (Lines 347-357)

Review Comments to the Author

Reviewer #1

In this paper, Kambara et al. reported the establishment of a de novo assembly pipeline for transcriptome data from the wild orchid Cypripedium. Using the contig set obtained by the established method, analyses of differentially expressed genes (DEGs) regarding mycorrhizal formation and maintenance were conducted. I agree with the authors’ idea that the de novo assembly pipeline established in this paper is effective and useful for the species for which genome sequences are not available such as Cypripedium to conduct RNA-seq. However, I have serious concerns about the statements of results and discussion based on the afterward transcriptome analysis because no biological replicates of samples were available. For example, the authors showed TPM value based on one sample data, but at least three biological replicates are generally demanded to demonstrate the gene expression levels (e.g., qRT-PCR). Generally, DEGs should be obtained based on reliable statistical analysis. Therefore, I am regret to say that GO enrichment analysis of the DEGs obtained from no biological replicate work has no biological meaning. I understand that the authors would like to validate the established pipeline by the afterward transcriptome analysis. However, I think the statement should be more restricted, considering the results obtained from insufficient replicates. Hence, I would like to propose that Fig. 4-6 are moved to supplemental information, and the statement regarding this part should focus on the validation. In particular, the discussion part (Line 389-421) is overdiscussed. Hence, I recommend that the contents of this part should be reconsidered and shortened as far as possible.

>> Response by authors

We’d like to thank Reviewer 1 for reviewing and for making positive, helpful comments about our manuscript. While we regret that we could not prepare more protocorms for RNA-seq, we agree with your concern about our transcriptome analysis; at least, three biological replicates should be prepared to compare gene expression and perform DEGs and GO enrichment analyses. According to suggestion, Fig. 4B, Fig 5, and Fig 6 were moved to supplemental information, and the statement regarding these results was modified. In the Result section, we focused on the validation of reference contig set by estimating expression levels to see the trend of expression levels of the OM interaction-associated genes in our interest (Lines 273-295). To further validate the use of our contig set, we also showed data of DEGs and GO analyses as an example of the downstream analysis of RNA-seq (Lines 300-319). Discussion parts regarding the results of DEGs and GO analyses were shortened, and we re-explained that the biological meaning cannot be discussed about the results obtained from DEGs and GO analyses because the analysis were performed without sufficient biological replicates (Lines 372-390)

In addition, I noticed that the abstract does not contain a conclusion at the end. Likewise, which content is the point the authors’ would like to claim to readers in this paper is not clear in the discussion part. Hence, please reconsider these contents and compositions.

>> Response by authors

Thank you for advice. We added a sentence for conclusion in abstract (Lines 33-35). In the Discussion section, we discussed about validities of contigs and considerations in the proposed pipeline, with minimal discussion of downstream analyzes performed by using the constructed contigs (Lines 372-390).

On the other hand, I think the validation of the assemblies using the data of genome-sequenced orchids is not enough because the authors compared only the number of predicted genes. The authors suggested the accuracy of assembling only based on the number of predicted genes, but I think that the accuracy should be shown based on the sequence identity. Thus, please add such analyzed data.

>> Response by authors

Thank you for your suggestion. To validate the assemblies constructed from RNA-seq data of genome-sequenced orchids, we calculated the sequence identity between assemblies and the protein-coding sequences in two orchid species. These results were included to the S3 Table and explained in the revised version (Lines 257-260, Lines 267-272).

I think the top five list summarized in Table 4 is also not required.

>> Response by authors

According to the suggestion, we deleted the Table 4 and original S2 Table (Top five assemblies for each evaluation when using one or two transcriptome data sets). As an alternative to not presenting these tables, we added some sentences about each evaluation (Lines 220-225, 241-246).

In addition, the assembled sequence data obtained in this study is not available, and only the unique gene IDs were shown in the manuscript. Considering that other researchers may verify or use the results obtained in this study, I strongly recommend that the representative assembled data should be deposited in public database database/website or submitted as supplemental data. I hope that the authors will revise the manuscript according to my comments.

>> Response by authors

We apologize for the incompleteness in Data availability. We have deposited our representative assembly data to the DDBJ Transcriptome Shotgun Assembly (TSA) database. We also added annotation information of the assembly contigs in S1 Table.

Minor comments:

Line 45: ‘orchid mycorrhiza fungi’ should be ‘orchid mycorrhizal fungi’.

>> Response by authors

We corrected the word ‘orchid mycorrhiza fungi’ to ‘orchid mycorrhizal fungi’. (Line 47)

Line 64: ‘et al’ should be ‘et al.’ (same afterward)

>> Response by authors

Thank you for your advice. We corrected all ‘et al’ to ‘et al.’ in revised version.

Line 72: Words at the beginning of a sentence are not abbreviated. (same afterward)

>> Response by authors

Thank you for your advice. We corrected them.

Line 73: Considering the content of previous sentence, ‘However’ is not a suitable word for connecting.

>> Response by authors

We changed the word ‘However’ to ‘On the other hand’. (Lines 75-76)

Line 108: The expression of ‘12-14 weeks’ is not suitable to show the timing to harvest each sample because two weeks range is so different. Please reconsider and show it for each sample.

>> Response by authors

Sorry for the inappropriate description. ’for 14 weeks’ is correct, we revised so. (Line 115)

Line 109: Please state the details about the strains WO97 and FT061. The current statement is not enough to understand what the authors used.

>> Response by authors

Thank you for advice. We included additional statement about WO97 and FT061. (Lines 117-119)

Line 226: What is ‘mapping rate’? I could not get its meaning. (same afterward)

>> Response by authors

Thank you for advice. We defined the term ‘mapping rate’ in Method section (Lines 153-154) and Table 1.
